# Audiological and Subjective Benefits in a Child with Microtia and Atresia After Sequential Bilateral Implantation with Active Bone Conduction Devices: A Case Study

**DOI:** 10.3390/children11111285

**Published:** 2024-10-24

**Authors:** Katarzyna Cywka, Anna Ratuszniak, Piotr Henryk Skarżyński

**Affiliations:** 1 Otorhinolaryngosurgery Clinic, World Hearing Center, Institute of Physiology and Pathology of Hearing, 02-042 Warsaw, Poland; 2 Department of Teleaudiology and Screening, World Hearing Center, Institute of Physiology and Pathology of Hearing, 02-042 Warsaw, Poland; 3 Institute of Sensory Organs, 05-830 Kajetany, Poland

**Keywords:** active bone conduction implant, hearing implant, Bonebridge, bilateral microtia, conductive hearing loss

## Abstract

Background: With bilateral hearing loss, the main problems for the patient are speech understanding in noise and, especially in asymmetrical hearing loss, an inability to correctly localize sound sources. There are multiple methods of treatment and rehabilitation for people with conductive hearing loss, and one of them is to use an active bone conduction implant. This case study is designed to evaluate the auditory benefits and sound localization accuracy with active bilateral bone conduction implants—in comparison to unilateral ones—in a patient with congenital bilateral conductive hearing loss caused by a congenital malformation. We assess subjective and audiological benefits (functional, directional hearing, speech comprehension in quiet and noise). Case report: This study describes the results in a 15-year-old patient with bilateral congenital malformation of the outer ears and associated conductive hearing loss who was treated with two Bonebridge active bone conduction implants. Speech recognition ability, hearing thresholds, and sound localization were tested under three conditions: unaided, unilateral on the right-side, and bilateral on both sides. The patient filled in an Abbreviated Profile of Hearing Aid Benefit questionnaire (APHAB) to evaluate limitations in daily life caused by hearing impairment. The results show an improvement in free-field hearing thresholds and the ability to discriminate speech, both in quiet and in noise after implantation. Subjectively, the patient had significantly fewer problems with two implants than with one (or with no implant) in terms of hearing in everyday situations. Conclusions: Unilateral use of the Bonebridge device in a patient with congenital bilateral conductive hearing loss did not provide full benefits. However, bilateral implantation improved speech understanding in noise and sound localization.

## 1. Introduction

Hearing loss in children can be congenital or develop later in childhood. Approximately 1–2 per thousand have mild to moderate bilateral or unilateral hearing loss, and 1.1 to 1.7 infants per thousand have permanent bilateral severe or profound hearing impairment [1]. The causes of congenital hearing loss can vary, including congenital deformities of the ear. One of these congenital defects is microtia and atresia of the external auditory canal. Microtia is a congenital condition characterized by the underdevelopment or absence of one or both ears. It can vary in severity, affecting the external ear’s size, shape, and structure. Microtia is often associated with the absence of an external auditory canal or a canal stenosis. Microtia is classified into four grades, ranging from mild underdevelopment to the complete absence of an external ear (anotia). Microtia usually occurs due to abnormal ear development during the first trimester of pregnancy and affects about 0.1 to 0.5 per thousand births, although rates vary by ethnicity. Microtia usually affects one ear but can affect both. The disorder is more common in males than females. Approximately 90% of cases affect one ear (more often the right ear) [2]. Approximately 80–90% of patients with pediatric microtia with atresia present with conductive hearing loss [3].

Hearing loss can occur in one or both ears, with bilateral hearing loss being either symmetrical or asymmetrical. A patient with bilateral hearing loss typically has problems with speech understanding, sound localization, and communication. In all likelihood, impaired localization plays a more significant role in speech communication difficulties than is usually thought. There is a direct correlation between the skill to localize sounds and the ability to hear speech in noise [4]. In difficult listening environments, with hearing impairment, localization plays an important role in following group conversations. When a conversation moves from one speaker to another, the listener needs to immediately locate the new speaker, for otherwise he or she will miss part of conversation, reducing comprehension [5].

In congenital hearing impairment, the main problem is curtailed auditory development that hinders the early acquisition of speech and verbal communication [3]. Not all patients are able to compensate for their hearing loss using air conduction hearing aids. In the case of microtia with atresia, a common difficulty is an inability to place the hearing aids in the underdeveloped ear. In such cases, various types of bone conduction (BC) devices can amplify the sound signal, bypassing malformed structures at the level of the outer and/or middle ear. Options can be non-surgical (a BC device on a soft band, on an adhesive adapter, or on spectacles) or surgical (a bone-anchored hearing device or a BC implant). Up to the age of five, non-surgical solutions are recommended, as some surgically implanted devices are only FDA-approved for children five years or older. Since it is important that the child receives help as early as possible, BC hearing aids on a soft band or adhesive adapter are the recommended first option [3]. Many studies have shown that patients rate the sound quality and performance of implantable devices more highly than those of non-implantable devices [6,7,8]. Patients appreciate wearing comfortable devices, improved speech understanding, and natural sound perception. Each case should be considered individually. The choice of a particular device depends on the results of the hearing test, the anatomical conditions, and the patient’s general health. For children with mixed or conductive hearing loss, only a few implantable BC devices are available on the market. The available devices vary in both visual and functional terms. Key aspects are the power of the device and the method of placement. One solution is the Bonebridge transcutaneous active BC implant. For full benefits, a patient with bilateral hearing loss should use the hearing implants binaurally. Healthcare procedures in different countries do not always allow for the surgical placement of two implants at the same time. This study shows that sequential implantation can provide benefits although it is “stretched” over time.

The aim of this study was to evaluate hearing and speech discrimination, sound localization ability, and subjective assessments of a patient with bilateral microtia and atresia who underwent sequential bilateral Bonebridge implants.

### Device Description

The Bonebridge system (BB) (Med-El, Innsbruck, Austria) was introduced to the EU market in 2012 and in 2014 was approved for children over 5 years of age [9,10]. The BB consists of an internal part (BCI 601/BCI 602 implant) with a BC floating mass transducer (BC-FMT) which is surgically implanted into the skull in the transmastoid, retrosigmoid, or middle fossa regions and an audio processor, which is the external part. The audio processor worn on the skin captures sound and sends the information through the coil to the internal part, which when placed in the bone transmits the vibrations directly to the inner ear. The BB is intended for children over 5 years old and adults. The indications recommended by the manufacturer are mild to moderate conductive or mixed hearing loss. Average hearing threshold levels at 500, 1000, 2000, and 4000 Hz (PTA4) should be better than or equal to 45 dB HL.

Individual aspects regarding implantation also need to be considered. Not only are audiological criteria assessed, but also the person’s medical history and anatomy, which should allow appropriate placement of the BB implant (as determined by a CT scan). Other important factors include the absence of central auditory disorders, emotional and psychological stability, and realistic expectations of the implant’s benefits and limitations [11].

The next generation of active bone conduction implants, the Bonebridge BCI 602, was launched in 2019. The main difference between the BCI 602 and BCI 601 implants is mainly in the design. In particular, the shape of the transducer and its size have changed—the thickness of the BC-FMT has been reduced from almost 8.7 to 4.5 mm. This is a significant advantage and creates opportunities for patients—especially children—who could not use the first-generation implant due to anatomical limitations. The BCI 602 version has the same power output, but requires a drilling depth almost half that of the BCI 601 implant [12].

In the first-generation BCI 601, a Samba 1 audio processor was used, while the second-generation BCI 602 uses Samba 2. The two devices are distinguished by the level of technological advancement in terms of advanced signal processing functions, housing design, software, and remote-control tools. The sizes, weight, type of power supply, and aesthetics of both devices do not differ significantly.

## 2. Case Report

This investigation was designed as a case study. The study protocol was approved by the Institutional Review Board of the Institute of Physiology and Pathology of Hearing (IFPS:/KB/07/2020) and complied with the Declaration of Helsinki.

This case study describes a patient with bilateral conductive hearing loss who underwent BB implant surgery at the World Hearing Center in Poland between 2018 and 2022. He was a 15-year-old boy with congenital bilateral aural atresia and microtia type 1. He had been using bilateral bone hearing aids on a soft band since he was 2 months old.

After undergoing a diagnostic process, the patient was qualified for a BC implant. Preoperative MRI imaging showed bilateral atresia of the external auditory canal. The mastoid process had an air cell structure. There was a dysplastic, deformed malleus and incus, although the stapes were normal. Topography of the facial nerve canal was normal. The osseous labyrinth had developed normally, without any sign of obliteration. In summary, the anatomical conditions were adequate for a BB implant.

**Figure 1:**
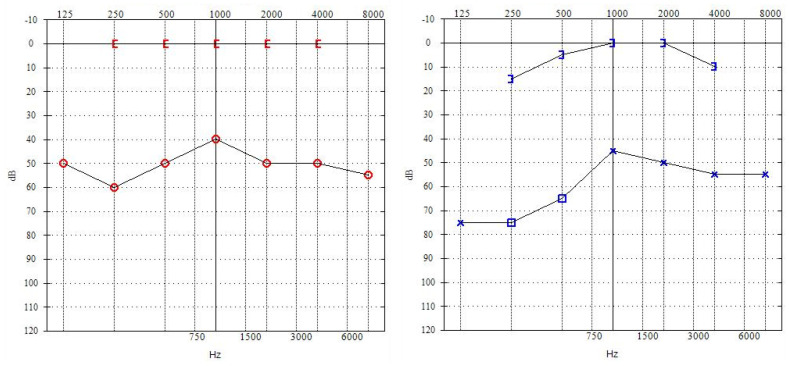
Results of pre-implantation pure-tone audiometry.

The BCI 601 implant was implanted first in the right ear in March 2018. Figure 1 shows the result of pure-tone audiometry performed on the eve of the implantation. BB implantation proceeded without complications. The BC-FMT was placed at safe distances from the external auditory canal, dura, and sigmoid sinus. At 4 weeks after surgery, the Samba 1 sound processor was programmed based on pure-tone thresholds, vibrogram, and the patient’s subjective feedback.

Due to the benefits and patient satisfaction obtained with the first implant, a qualifying procedure for implantation of the BB in the left ear was carried out in 2021. In March 2022, the patient was implanted with a BCI 602 in the left ear. Some 5 weeks after surgery, the Samba 2 audio processor was programmed in the same way as the first device. No complications were observed either after the first or second surgery.

The following describes the results of the treatment. Hearing thresholds, sound localization, and speech discrimination were tested 12 months after the first, and again 12 months after the activation of the second BB, under three conditions: U, unaided; R, unilateral BB aided on the right side; and B, bilateral (aided on both sides). During the audiological test, the processor was set up in omnidirectional mode. Following implantation, the patient was advised to undertake auditory training in everyday life, including practicing sound detection, identification, and classification. Follow-up visits to fitting the processor to current subjective impressions and to monitor benefits took place 1, 3, and 12 months after activation. Psychological support was available to the patient at each clinic visit.

### 2.1. Audiological Tests

Sound-field hearing thresholds (SFHTs) were measured using warble tones at 0.5, 1, 2, and 4 kHz in a soundproof room via loudspeakers 1 m in front of the patient. The Word Recognition Score (WRS) in quiet was measured using the Demenko & Pruszewicz Polish monosyllabic word test at 65 dB SPL presented via the loudspeaker. The SRT in noise was assessed with both signal and noise presented from the front (S0N0); the noise level was fixed at 65 dB SPL and the signal level was changed adaptively. Speech reception thresholds (SRTs) in noise were assessed using the Polish Sentence Matrix Test (PSMT). The PSMT was used to measure the intelligibility of speech presented against a background noise, and it consists of five columns containing 10 nouns, 10 names, 10 verbs, 10 numerals, and 10 adjectives. The SFHT, WRS, and SRT in noise were assessed 12 months after the first implantation and again 12 months after the second.

### 2.2. Sound Source Localization

**Figure 2:**
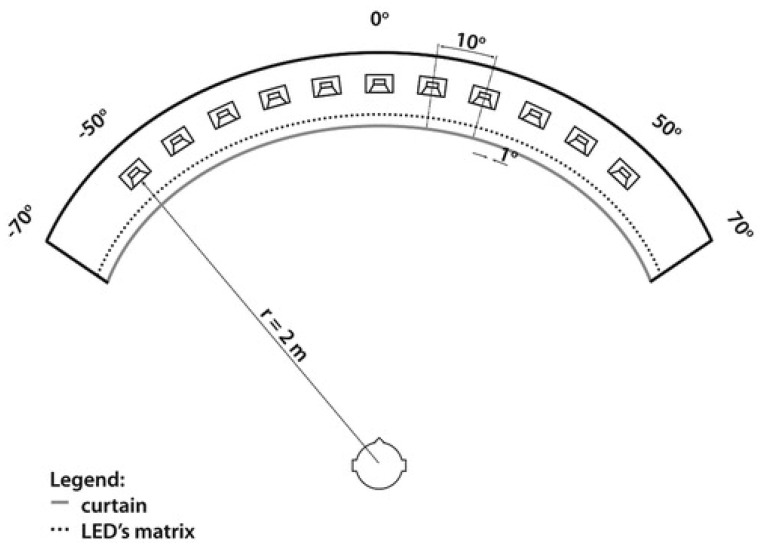
Arrangement of 11 loudspeakers and 141 LEDs for testing sound localization [13]. Reprinted/adapted with permission from Ref. [Copyright year, copyright owner’s name Skarzynski, H.; Lorens, A.; Kruszynska, M.; Obrycka, A.; Pastuszak, D.; Skarzynski, P.H. The hearing benefit of cochlear implantation for individuals with unilateral hearing loss, but no tinnitus. *Acta Oto-Laryngol.* **2017**, *137*, 723–729]. 2017, Skarzynski H.

Sound localization testing was performed in an anechoic chamber using a custom-made system of eleven loudspeakers fixed in a semicircle 2 m in diameter, as shown in Figure 2. The speakers were located from −50° to 50° in the frontal horizontal plane (left–right), with each loudspeaker separated by 10°and covered by a curtain. The patient sat at the center of the semicircle and, with the use of a control pad, lit up one of 141 LEDs (separated by 1°) located underneath the loudspeakers and which corresponded to the perceived direction of the sound (which could range from −70° to 70°). For the test, eleven different environmental sounds were used. The sounds were presented randomly twice. The subject was tested twice with the BB on the right side (R) and twice with the BB on both sides (B).

The localization test was conducted 12 months after the second implantation, first with unilateral BB aided on the right side (R), and once again bilaterally, with the BB on both sides (B).

### 2.3. Questionnaire Assessment

The patient’s subjective satisfaction and quality of life with the unilateral and bilateral BB were assessed using the Abbreviated Profile of Hearing Aid Benefit questionnaire (APHAB) [14]. APHAB measures subjective hearing impairment on four different subscales relating to different listening situations. The subscales are EC, ease of communication; BN, background noise; RV, reverberation; and AV, aversiveness. The global score is the mean score of the first three categories. Interpretation of the APHAB (Abbreviated Profile of Hearing Aid Benefit) questionnaire involves analyzing scores on various subscales to understand individual problems in different hearing situations. Each subscale score ranges from 0 to 100, where 0: means maximum benefit (no difficulty), 100: means maximum difficulty (severe problems). A higher score indicates greater difficulty or more significant challenges in everyday situations. By comparing results before and after hearing implants, the overall benefits and effectiveness of the intervention can be assessed. The questionnaire was completed before implantation, 12 months after the first implantation, and 12 months after the second implantation.

### 2.4. Sound-Field Hearing Thresholds (SFHTs) and Word Recognition Scores (WRSs) Results

**Figure 3:**
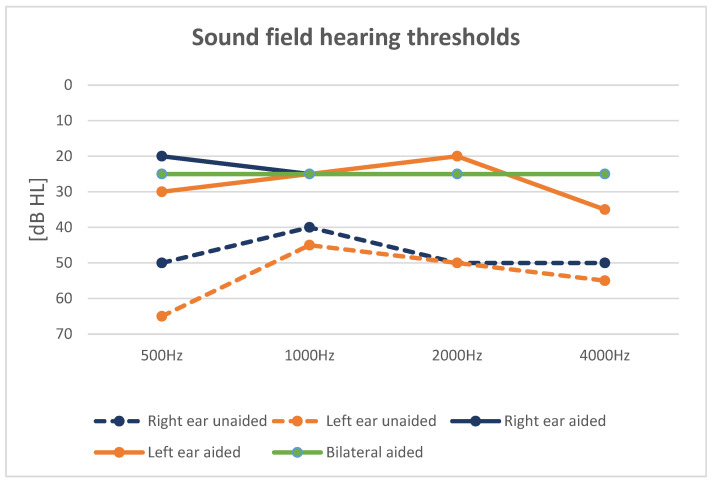
Results of free-field audiometry.

The unaided SFHT was 47.5 dB HL (average hearing thresholds for frequencies of 500, 1000, 2000, and 4000 Hz) for the right ear, and 53.75 dB HL for the left, while the aided hearing threshold for the right-ear aided condition was 23.75 dB HL, and for the left-ear aided condition 27.5 dB HL. The bilateral-aided SFHT was 25 dB HL. Results across frequencies from 500 to 4000 Hz are presented in Figure 3.

The unaided WRS at 65 dB was 10% for the right and left ears, while the aided WRS was 90% in the R-aided condition and 100% in the B-aided condition. There was no difference in WRS between right-ear and left-ear aided conditions. The highest WRS was observed in the bilaterally aided condition.

SRTs in noise decreased significantly from 14 dB SNR (signal-to-noise ratio) for the unaided condition to 1 dB SNR in the right-aided condition, and −4.5 dB SNR in the bilaterally aided condition.

### 2.5. Sound Source Localization Results

Root-mean-square error (RMSE) in sound localization tests in the R condition (version number of the software: DirHearing v. 2.1.6) was 39.5°, and the corresponding figure with B was 29.1°. The localization benefit was estimated by subtracting the RMS error in the B condition from that in the R condition and was found to be 10.4°.

### 2.6. Questionnaire Assessment Results

**Figure 4:**
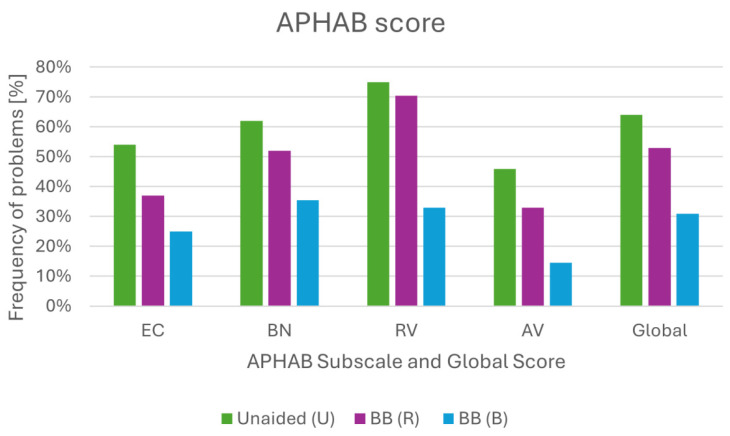
Aided and unaided scores of the APHAB questionnaire: EC, ease of communication; RV, reverberation; BN, background noise; AV, aversiveness.

The questionnaire was conducted before surgery (unaided, U); 12 months after implantation in the right ear (R); and 12 months after implantation in the left ear (B). The results indicated fewer hearing loss problems when the implants were used binaurally. The most significant decrease in problems was observed in the area of speech understanding within the reverberation and background noise subscales. Figure 4 shows the scores obtained on all subscales, comparing unaided (U), unilateral (R), and bilateral (B) use of the implants. The smallest significant difference between unilateral and bilateral use (just 12 p.p.) was noted on the EC subscale. When using only one device, the patient had no major problems in communicating in simple acoustic situations. The largest difference was obtained on the RV subscale (37.5 p.p.). On the AV scale, the difference between R and B was 18.5 p.p. The global score for unaided conditions was 64%, when using only one implant (R) it was 53%, and it was 31% for implants on both sides (B).

## 3. Discussion

Since the introduction of Bonebridge into clinical practice, many publications have appeared regarding its benefits in children, most of whom exhibit congenital defects [9,15,16,17,18,19,20,21,22]. The results in the present case are consistent with the results reported in the literature on the use of an active implant in difficult surgical cases, especially in those involving congenital defects such as microtia with atresia. It is generally reported that the Bonebridge system provides good and stable audiological benefits for patients suffering from mixed and conductive hearing loss, including children. In a group of 15 pediatric patients with bilateral microtia with atresia and treated with the Bonebridge system, Bravo-Torres et al. [15] achieved average hearing thresholds (free-field) 1 month after activation of 25.2 dB. Similarly, Zernotii et al. [23] reported an average level of 19.2 dB in a group of 14 children and adolescents (3–17 years old), including 7 implanted binaurally, which is comparable to the results obtained here. In terms of speech recognition, Bravo-Torres et al. reported an average of 96% 1 month after activation, similar to Zernotii’s 97%, while in our study the patient reached 100% speech discrimination. Fan et al. [24] investigated a series of 12 pediatric patients with external auditory canal atresia. The mean unaided PTA was 55.25 dB HL, while the mean PTA aided with Bonebridge was 21.25 dB HL; the unaided speech discrimination score at 65 dB presentation level was 46.0%, and it improved to 94%. Finally, the results of a multicenter study, involving both children and adults, reported stable benefits over a 36-month follow-up [25]. Most articles in the literature report results of unilateral implantation.

With Bonebridge, hearing in noisy environments improved significantly compared to with no hearing aid. Sound localization also improved when two devices were used instead of one. This is noteworthy, because bone conduction hearing aids stimulate both cochleas. The improvement in quiet with bilateral fittings can be attributed to increased stimulus levels: there is diotic summation of signals from each side, which improves dynamic range. Priwin et al. reported that users of bone conduction hearing aids received more benefit from bilateral stimulation if they had good cochlear function compared to those with poorer cochlear function [26]. Similarly, the research of Zeitooni and colleagues confirms the benefits of bilateral bone conduction stimulation in those with good cochlear function, and these Swedish workers consider it a practical option [27].

A number of other studies have reported a positive outcome of bilateral bone conduction hearing devices compared to unilateral ones, and a systematic review by Colquitt et al. summarizes the benefits of such devices in patients with bilateral hearing loss [28]. In related work, Dutt et al. compared the benefits of bilateral implantation with unilateral amplification in eleven patients who used their second BAHA (bone-anchored hearing aid) for at least 12 months [29]. With both conditions (bilateral and unilateral BAHAs), a 100% score was achieved on “speech in quiet” tests. With “speech in noise”, all eleven patients with bilateral implants scored better. Dutt and colleagues showed that patients fitted with bilateral BAHAs report better sound quality and they consistently prefer bilateral fittings to unilateral ones [29]. These results and conclusions correspond to those obtained in our work.

In terms of improved sound localization, there are a number of studies in patients implanted with bilateral bone conduction devices that have indicated binaural benefits, and to a limited extent, speech understanding in both quiet and noise [26,30,31,32,33]. In a group of 25 people bilaterally fitted with BAHAs, Bosman et al. demonstrated improved directional hearing and speech recognition in noise when the second BAHA was in use [31]: the SRT was 1 dB SNR with one implant and −5.5 dB SNR with both. den Besten et al. [33] studied the lateralization and localization of sounds in 11 children with bilateral conductive hearing loss when listening with either one or two percutaneous bone conduction devices; they concluded that the children lateralized sounds better when listening with bilateral devices. Despite the sub-optimal results with sound localization, their study underlines the merits of bilateral application of bone conduction devices in such children [33]. Fan et al. studied the effects of bilateral bone conduction stimulation in children with microtia–atresia using a Bonebridge implant and the Adhear device (an adhesive bone conduction hearing aid from Med-El); they reported that functional hearing and sound localization were better with bilateral devices than with unilateral ones [34].

It is difficult to compare our localization results with the results from other work because the methods differ significantly from author to author (different set-ups, number and locations of loudspeakers, sound stimuli, listener responses, etc.). The problems associated with comparing localization findings were noted at a recent meeting of the Hearring group. The consensus was reached that the classification should follow the categories of the ICF (International Classification of Functioning, Disability and Health). The methodology enables the results obtained with a given set-up to be linked back to ICF categories and compared with others. In their paper “Towards a consensus on an ICF-based classification system for horizontal sound-source localization”, Mertens and colleagues introduce a 5-level classification system to describe difficulties in locating a sound source (0, no problem, to 5, complete problem) [35]. According to this system, in our case the result with one Bonebridge implant (R) was 39.5 and was in the “severe problem” range; with two implants (B), the figure was 29.1, indicating a “moderate problem”. The change by one category indicates a clinically significant improvement.

To the authors’ knowledge, there are relatively few publications describing bilateral implantation using active bone conduction implants [32,36,37,38,39]. Dusu et al. describe a pediatric case involving bilateral Bonebridge devices implanted at the same time in the left middle cranial fossa and the right sinodural angle. They report that the devices were well tolerated and gave significant improvement in hearing [37]. In turn, Chen et al. [36] report the results of hearing tests and sound localization in a group of eight patients with congenital microtia with atresia who received bilateral Bonebridge devices implanted simultaneously. The authors report an improvement in the speech reception threshold in noise with bilateral fitting compared to unilateral fitting; bilateral fitting also gave better sound localization. They also suggest that sound localization with bilateral devices can be improved through long-term training [36]. Although the results of simultaneous implantation have been shown to be good, our case shows that sequential implantation also has measurable benefits. Although it takes more time to achieve the full benefits, it provides access to binaural hearing with all its advantages. In situations where simultaneous implantation is not possible or not recommended, it is advisable to plan a sequential procedure.

There are several studies supporting our finding of increased life satisfaction from bilateral fitting [39,40]. One study conducted in 2002 looked more specifically at patient satisfaction with a second device for bone conduction. A higher degree of patient satisfaction and improved quality of life after a second BAHA implantation compared to a single device has been reported [29], mainly in the form of less difficulty in daily functioning.

Our work is a single-case study and so we cannot draw broader conclusions. For strong evidence of the effectiveness and efficiency of bilateral active bone conduction implants, research on a group of patients is needed.

## 4. Conclusions

Early identification, treatment, and medical evaluation of hearing loss in children is, regardless of age of onset, essential to allow normal auditory, language, and social proficiency to develop. Current clinical practice guidelines and protocols for pediatric hearing aid fitting recommend managing these conditions on a case-by-case basis. Binaural hearing is an important auditory function that allows individuals to achieve the best possible speech recognition in complex situations (especially in difficult acoustic conditions). The use of a unilateral hearing device in a patient with congenital bilateral conductive hearing loss is of benefit, but it does not provide a full range of benefits, especially in terms of localization and understanding of speech in noise. Bilateral implantation improves both these aspects. In situations where simultaneous implantation is not possible, a sequential procedure may be the answer. More research is needed to confirm the efficacy of bilateral bone conduction implants for bilateral conductive and mixed hearing loss.

## Data Availability

Dataset available on request from the authors.
